# Hepatitis Delta Virus Infection: An Overview

**DOI:** 10.3390/pathogens14090899

**Published:** 2025-09-06

**Authors:** Vitor Duque, Diana Duque

**Affiliations:** 1 Faculty of Medicine (FMUC), University of Coimbra, 3000-548 Coimbra, Portugal; 2 Department of Marketing, HEC Lausanne (UNIL), 1015 Lausanne, Switzerland; dianacduque@gmail.com

**Keywords:** hepatitis delta, epidemiology, superinfection, coinfection, treatment

## Abstract

The burden of hepatitis delta virus (HDV) infection is currently unknown and may affect 12 to 72 million people distributed across various hot spots in different regions of the globe. Screening for antibodies to HDV infection in patients positive for the hepatitis B surface antigen (HBsAg) is generally available in most parts of the world, but systematic testing for HDV is needed. Chronic HDV infection is associated with a higher risk of progression to cirrhosis, liver failure, and hepatocellular carcinoma compared to hepatitis B virus (HBV) mono-infection. Bulevirtide is the recently available treatment against hepatitis delta. The results of efficacy studies and new drugs (lonafarnib) are under discussion. New therapeutic strategies are in development, revealing a critical need for valid next-generation treatments to cure HDV.

## 1. Introduction

The hepatitis delta virus was first identified by Rizzetto and colleagues in 1977 in Italy in the course of examining human liver tissue to detect the hepatitis B core antigen [1]. Viruses similar to HDV have been identified in several animals (including invertebrates and termites); they have biologic features similar to the viroids of plants, indicating that the earliest form of HDV might have emerged several million years in the past [2]. Currently, it falls within the Deltavirus genus of the Kolmioviridae viral family [3].

This article seeks to provide an updated, detailed review of HDV infection, focusing on recent advancements in the viral life cycle and potential emerging therapeutic strategies. By synthesizing the latest research, this review seeks to enhance understanding of HDV infection and guide future research and clinical practices.

## 2. Structure and Replication

The HDV is the smallest viral pathogen infecting humans (35 to 37 nm in diameter) and is described as a “satellite,” “incomplete”, or “defective virus” because it depends on HBV for cell entry and to be released from infected hepatocytes [1,3].

The hepatitis delta virion consists of an external lipoprotein-based envelope that generally includes three variants of the surface antigen found in the hepatitis B virus: large surface protein (L-HBsAg), middle surface protein (M-HBsAg), and small surface protein (S-HBsAg). Inside there is a ribonucleoprotein (RNP) structure containing multiple copies of the hepatitis delta antigen (HDAg), complexed with a single molecule of a single-stranded, negative-sense, circular ribonucleic acid (RNA) genome of approximately 1.7 kb in length. The genome does not encode enzymes involved in replication or envelope proteins; instead, it only encodes a small, non-enzymatic protein, the delta antigen (HDAg). Two different forms of DAg are produced: small (S-HDAg) and large (L-DAg) [4]. Host cell entry by HBV involves weak binding to heparan sulfate proteoglycans (HSPGs) [5], followed by strong affinity and specific attachment of the pre-S1 domain of L-HBsAg of the envelope at the hepatocyte entry receptor Na-taurocholate cotransporting polypeptide (NTCP) [6]. HSPGs are glycoproteins found at the surface of almost all cells. HSPGs act as receptors and coreceptors, play key physiological roles, and have been associated with the attachment of multiple viruses (including dengue virus, herpes simplex virus, and human papillomavirus) [5,6]. NTCP mediates the sodium-dependent transport of bile acids across the basolateral membrane of hepatocytes. The dysfunction of NTCP causes impaired bile acid transport, resulting in abnormally high concentrations of bile acids in the plasma [5,6]. The binding of viral particles to the cell surface induces cell activation and may lead to virus internalization via various endocytic mechanisms in many cases. Entry into the permissive cell occurs through a clathrin-mediated endocytosis and RNP complex (enclosing a RNP made up of several copies of HDAg and one copy of the viral genome) transported into the nucleus [6].

Viral replication is confined to hepatocytes and propagates through the liver via both extracellular dissemination and pathways mediated by cell division, facilitating widespread infection. The most established route is extracellular or HBsAg-dependent virion propagation, where hepatitis delta virions interact with NTCP receptors, enabling cell-to-cell transmission. In the other cell division-mediated pathway or HBsAg-independent route, the virion survives cell division and is transmitted to both daughter cells [7]. After being internalized by endocytosis, enveloped viruses continue along the endocytic pathway, sequentially trafficked through early endosomes, late endosomes, and endo-lysosomes, until they reach the cytoplasm, where uncoating and subsequent replication occur. Dependence on low pH is the primary determinant for triggering fusion for the majority of enveloped viruses taken up via endocytosis. The RNP complex is shuttled to the nucleus, where DAg mRNA transcription and HDV RNA replication start. Replication depends on host RNA polymerases [4,5,8].

Three HDV RNA species are detected in humans: a 1.7 kb genomic RNA present in virions, a complementary antigenomic RNA of positive sense with the same size, and a smaller 0.8 kb antigenomic polarity mRNA in the liver; the mRNA carries a single open reading frame responsible for translation of HDAg isoforms. S-HDAg is essential for both the initiation and the sustained progression of replication. L-HDAg isoform demonstrates distinctive functional properties. It has the capacity to inhibit genome replication; it possesses a c-terminal extension that contains a farnesylation signal enabling the addition of a farnesyl group by the cellular farnesyltransferase to facilitate virus assembly in a process called prenylation; the secretion of complete HDV virions and virus-like particles (VLPs) consists solely of L-HDAg and S-HBsAg. So, replication or packaging signals may depend on the relative concentrations of both S- and L-HDAg [4,5,8]. HBsAg proteins originate from transcripts of covalently closed circular HBV deoxyribonucleic acid (HBV DNA), together with transcripts derived from linear HBV DNA incorporated into the host genome [9]. S- and L-HDAg bind to the newly produced RNA. Both HDAgs possess the N-terminal S-HDAg segment and are capable of binding genomic and antigenomic HDV RNA to assemble RNPs. Viral RNPs are formed and exported to the cytoplasm [4,5,9]. When HBsAg is co-expressed in the same cell, prenylated L-HDAg recognizes a hydrophobic motif located within the cytosolic loop of the S-HBsAg envelope protein. HBsAg expression in RNP-containing cells alone is sufficient to mediate HDV secretion. By incorporating L-HBsAg, the particles gain infectivity and support transmission [9]. HDV relies on HBV for viral packaging, infectivity, transmission, and inhibition of host immunity.

## 3. Global Epidemiology of Hepatitis Delta Virus and Viral Genotypes

In 2022, the World Health Organization (WHO) estimated that 5% of HBV-infected patients globally—over 14 million people—were also HDV-infected. However, various published meta-analyses have estimated the worldwide number of patients infected with HBV-HBD infection as varying from 12 million to 72 million people, meaning the prevalence varies from 0.16% to 0.98% [10]. Countries with high HBV endemicity may not necessarily exhibit high HDV endemicity, but several geographical hotspots of high HDV prevalence are dispersed across the world. Some areas of the globe, like Greenland and the Amazon Basin, which host multiple indigenous populations and are where several subtypes circulate, exhibit a high prevalence of HD despite not being a highly endemic region for HBV [11,12]. Among HBsAg-positive individuals, the global prevalence of HDV is estimated to fall between 4.5 and 14.6% [11,12].

HBV immunization programs, launched in the 1990s in high-income countries, have significantly reduced the prevalence of both HBV and HDV infections. At present, the prevalence of both infections is greater in older generations because younger populations are protected against HBV, and secondarily against HDV by HBV vaccination programs. In Europe, the decline has been most marked in the youngest people [12]. However, the observed drop in HBV and HDV in the native European population may be counterbalanced by the introduction of new infections through immigration from regions where HDV remains endemic, as is happening in Italy and is also being reported in other wealthy nations [12,13]. Prevalence among HBsAg-positive individuals is the highest in Asia, particularly in Mongolia, in some parts of Eastern Europe such as Romania and the Republic of Moldova, and in countries in Western and Central Africa, including Tunisia, Benin, and Niger. The countries carrying the greatest overall disease burden are China, Pakistan, and Brazil [4,13].

Among people living with human immunodeficiency virus (HIV) who do not inject drugs, the prevalence is comparable to that observed in human immunodeficiency virus (HIV)-negative populations [14]. To examine epidemiological trends of HDV across Europe, a recent analysis of data from a large HIV cohort collaboration study (the Swiss HIV Cohort Study and EuroSIDA) showed a similar prevalence across countries to that observed in the general population without HIV who do not inject drugs [14]. But a higher prevalence was observed among people who inject drugs (PWID) across Europe, which may be as high as 50.5% [4]. Coinfection with HDV is linked to an elevated risk of mortality, hepatic-related death, and hepatocellular carcinoma [14].

Eight genotypes (one to eight) are circulating worldwide, with 81% to 89% sequence homology, and many subtypes have been identified. The geographic distribution of HDV genotypes is as follows: genotype 1 (Europe and the USA), genotype 2 (Asia and the Middle East), genotype 3 (Amazon), genotype 4 (China and Taiwan), and genotypes 5, 6, 7, and 8 (Africa, South America, and Europe) [4,15]. Genotype 3 is the most pathogenic and causes severe forms of liver disease [4,15].

## 4. Patterns of Transmission

HDV is most commonly transmitted through the parenteral route. Vertical transmission from mother to child is described as being exceptionally rare [16]. Transmission via sexual intercourse is also infrequently observed. In most countries, heterosexual exposure may be an important route of HDV transmission among populations in areas where HBV is endemic [17].

Transmitted infection may follow two patterns: coinfection or superinfection. In HBV-HDV coinfection, both viruses infect individuals at the same time, leading to acute self-limited hepatitis in more than 90% of cases, ending in complete viral clearance. However, less than 10% of these cases progress to chronic HDV infection [4,5,8]. After an incubation period of 3 to 7 weeks, an acute HDV infection may follow; patients may present with either icteric or non-icteric acute hepatitis with nonspecific influenza-like symptoms, such as nausea, tiredness, appetite loss, and lethargy, along with increased aminotransferase levels. In uncommon instances, it can lead to severe acute hepatitis or even fulminant hepatitis [4,5,8]. In superinfection, HDV infects individuals who already have pre-existing HBV infection. It causes a clinically severe acute hepatitis, and up to 80% of cases progress to chronic hepatitis B-delta infection [4,5,8]. However, in mild cases, patients may have nonspecific symptoms [4,5,8].

In chronic HDV infection, clinical outcomes have been described as worse than in HBV mono-infection, with risks for developing cirrhosis increased two- to threefold, three-to sixfold for hepatocellular carcinoma, twofold for hepatic decompensation, and also a higher risk of mortality (twofold) [4]. The presence of HDV RNA in the blood confers a 3.8-fold higher risk of adverse liver outcomes, but is lower among patients with undetectable HDV RNA viremia [18]. Treatment of chronic HDV infection often requires liver transplantation, a procedure that is used for treating end-stage liver disease, not for the cure of HDV infection. The grafted liver may also become infected. However, there is increasing evidence that a significant subset of patients have an indolent progression through the natural history of the disease. Up to 25% of the patients with chronically HDV-infected patients may have a progressive decline of 2 or even more log drops in HDV RNA blood load, with some of them reaching undetectability over a follow-up period of approximately 5 years [18].

## 5. Diagnosis

Limited access to HBV DNA testing and the necessity of attending multiple visits for sample collection and result reporting remain significant barriers to starting treatment after a positive HBsAg test. Worldwide, only a small proportion of HBsAg-positive individuals undergo testing for HDV infection (anti-HDV antibodies). To detect most of the patients with HDV infection, two testing strategies may be implemented, one based on testing for hepatitis delta only in HBV-infected patients at risk of acquiring HDV infection (risk-based approach) and the other based on testing all patients diagnosed with HBV for HDV infection (universal-based approach) [19].

A risk-based screening approach does not appropriately screen for HDV. Universal screening of HDV in HBV-infected patients is the appropriate approach to diagnose all patients for treatment. However, risk-based screening may be most useful when universal anti-HDV antibody screening is not feasible, such as in areas of limited resources or limited laboratory capacity. In these situations, screening for anti-HDV may be performed only in specific subsets of HBsAg-positive individuals, such as those born and living in HDV-endemic areas; patients with advanced hepatic disease, undergoing hepatitis B therapy who exhibit features suggestive of HDV infection, such as low HBV DNA alongside high alanine aminotransferase (ALT) levels; populations at higher risk for HDV infection, including hemodialysis recipients; people coinfected with hepatitis C virus or HIV; sex workers; PWID; and men who have sex with men [19].

A universal-based approach through double reflex testing—first for anti-HDV antibodies and subsequently for HDV RNA following a positive HBsAg result—is what is recommended by the WHO to promote diagnosis and link patients to care and treatment [19]. Double reflex HBV-HDV testing can be applied through an algorithm in the laboratory (laboratory-based reflex testing) or at the point of care (clinic-based reflex testing). This may avoid the need for additional venipuncture and blood draws, increase the uptake of anti-HDV antibodies and also HDV RNA testing, reduce the number of clinic visits for blood sample collection and follow-up, and may be associated with improved outcomes across the cascade of care [20].

Implementing laboratory-based reflex anti-HDV testing represents a crucial element of establishing a universal testing strategy for all individuals with a positive HBsAg result. However, the diagnostic accuracy of HDV serological assays and nucleic acid amplification tests (NATs) still needs to be validated [19].

Diagnosis and characterization of HDV infection involves specific serologic testing combined with NATs. Serological markers available include the detection of HDAg together with anti-HDV antibodies of the IgM, IgG, and total classes. Generally, screening for chronic HDV infection is performed by detecting total or IgG anti-HDV antibodies [5,8]. The applicability of serological testing in children under two years of age is limited, as maternally derived, placentally transferred antibodies may persist until this age, leading to false-positive anti-HDV results. The presence of antibodies to HDVAg in an HbsAg-positive patient is a marker of previous exposure to hepatitis delta. These patients may have an active infection, either acute or chronic, if they have HDV RNA detectable in the blood. If HDV RNA remains repeatedly detectable in the bloodstream for longer than six months, a diagnosis of chronic infection is made. Individuals positive for HBsAg and anti-HDV antibodies but lacking detectable HDV RNA in blood may have experienced a past HDV infection. Individuals testing positive for anti-HDV antibodies should, if possible, undergo serum HDV RNA testing to ascertain the presence of active infection [5,8]. Commercially available assays exist for qualitative detection of anti-HDV antibodies. However, no international standard currently exists for their quantitation. Several commercially available kits for specific serologic testing have demonstrated good reproducibility of results across different laboratories. Also, while a number of HDV RNA assays are commercially available for clinical use, only a limited subset has been standardized [19].

According to WHO guidelines, NATs for the detection of HDV RNA should be standardized using the WHO HDV RNA reference standard, with the results expressed in IU/mL. These assays should possess a lower limit of detection of at least 100 IU/mL. Primers are recommended to target the ribozyme region, the most conserved region of the HDV genome, to ensure inclusivity across genotypes [19]. However, although HDV RNA assays consistently quantify HDV genotype 1 in plasma, they may underestimate or fail to detect the African genotype 1 and genotypes 5 to 8 [4,20].

The diagnosis of acute HDV-HBV coinfection is established by the detection of HBsAg and IgM antibodies against the hepatitis B core antigen (IgM anti-HBc), subsequently confirmed through the identification of HDV RNA and anti-HDV in the blood [5,8]. HDAg is transient and challenging to identify, but may appear early, while anti-HDV is a late marker in the bloodstream. Resolution of HBV/HDV infection is indicated by the appearance and persistence of hepatitis B surface antibody (anti-HBs) and anti-HDV with disappearance of HBsAg and HDV RNA [5,8].

Superinfection criteria for diagnosing chronic HDV rely on the sustained presence of HDV RNA for more than 6 months in a patient with previous markers of chronic hepatitis B infection [5,8]. HDV superinfection can present either as acute hepatitis or as an exacerbation of chronic hepatitis B. Diagnostic confirmation relies on the identification of HDV RNA and anti-HDV markers in those previously known to be HBsAg-positive, with IgM anti-HBc remaining negative. Serum HDAg may also be detected early but exhibits a brief duration. Anti-HDV appears late but persists in the blood. In HDV superinfection, HBsAg and HBV DNA levels may decrease in the blood, but in chronic infection, markers of both infections commonly remain positive for HBsAg, HBV DNA, anti-HDV, and HDV RNA [5,8].

## 6. Overview of Hepatitis Delta Treatment

Interferons (IFNs) are nonspecific inhibitors of viral replication and stimulate immune responses against HDV infection. Interferon alfa (IFN—α) and pegylated interferon (peginterferon) alfa-2a have been used off-label in several clinical trials. They are able to effectively inhibit HDV replication, but after 48 weeks of treatment, only up to 30% of patients may have a sustained virologic response (i.e., absence of detectable HDV RNA in serum for six months after discontinuation of treatment), and long-term relapses are common [4].

Pegylated interferon lambda may be an alternative; studies are ongoing, and it has been shown to have greater efficacy rates of virologic response and superior tolerability to interferon alfa at 24 and 48 weeks. A post-treatment response rate of 50% was observed in patients with low baseline viremia (≤4 log10) receiving a 180 mcg dose, suggesting that patients with low baseline viremia may be more likely to obtain non-detectable HDV viral load after treatment cessation, particularly in individuals infected with genotype 1 [20].

**Figure 1:**
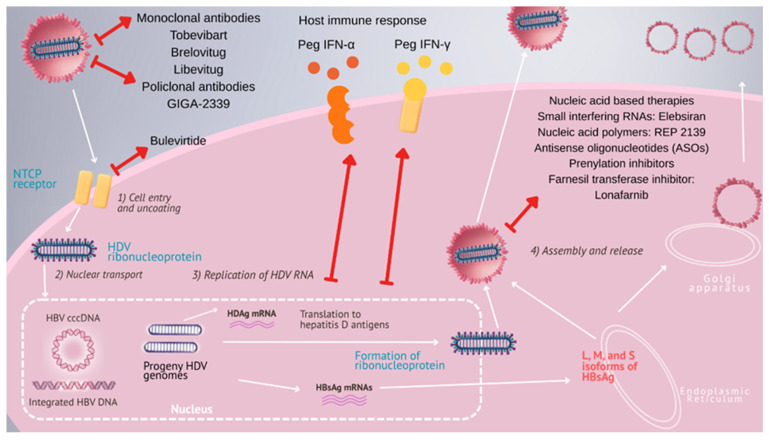
Targeting the hepatitis D virus life cycle: current and investigational antiviral strategies.

The optimal therapeutic endpoint is considered to be the clearance of HBsAg; however, this outcome has been infrequently attained with interferon or even with the newly proposed treatments [21]. New primary goals or primary endpoints to assess the efficacy of new drugs against hepatitis delta have been proposed based on the levels of viremia and the aminotransferase values: undetectable levels or a drop in viral load equal to or greater than 2 logs from the baseline and the biochemical response. However, the clinical meaning of these new goals of HDV treatment is not known [21]. Until recently, no officially approved therapy was available for chronic hepatitis delta (Figure 1, Table 1).

## 7. Small-Molecule Drug-Based Therapeutics

### 7.1. Bulevirtide

Bulevirtide—used alone—is currently the only formally approved therapy for chronic hepatitis delta. In July 2020, the European Medicines Agency approved marketing authorization for a 2 mg daily subcutaneous dose of the drug to be used in adults and children aged 3 years and older, weighing at least 10 kg, with chronic compensated hepatitis delta [21]. However, the most effective dose and treatment duration remain unclear. Bulevirtide represents a first-in-class peptide-based entry inhibitor targeting HBV and HDV infections. Hepatocyte bile salt uptake is mediated by NTCP on the basolateral membrane. HBV initially attaches to hepatocytes via low-affinity interactions with HSPG, followed by specific, high-affinity binding to NTCP, which facilitates viral entry. This interaction occurs through the myristoylated preS1 domain of the L-HBsAg binding to NTCP. The entry of HBV into hepatocytes is blocked by bulevirtide, a peptide derived from preS1 [21,22]. Marketing authorization was based on some published studies that show us what can be expected from treatment with bulevirtide, as monotherapy or combined therapy in the short and long term.

The MYR 301 trial, a long-term monotherapy study (48-week interim analysis, randomized trial, open-label, phase 3), evaluated patients with chronic hepatitis delta (with or without compensated cirrhosis). It compared bulevirtide at different doses of 2 mg or 10 mg for a total of 144 weeks, with a group of no bulevirtide for the first 48 weeks, followed by subcutaneous bulevirtide 10 mg per day for the subsequent 96 weeks. The primary endpoint at week 48 was a composite response, defined as undetectable HDV RNA or a decrease of at least 2 log10 IU/mL in the HDV RNA, together with normalization of the ALT levels. At week 48, a combined response was obtained in 45% of patients in the 2 mg group, 48% in the 10 mg group, and 2% in the 2 mg in the control group. ALT levels normalized in 51% to 56% of patients in the treatment groups versus 12% in the control group. Undetectable RNA was achieved in 12% of the group of patients receiving the 2 mg and in 20% of those patients receiving the 10 mg. There was no decrease in HBsAg levels of at least 1 log10 IU/mL, and no one became undetectable in both groups by week 48 [22]. However, longer bulevirtide monotherapy for 96 weeks resulted in continued benefits in overall, virologic, and biochemical outcomes, along with liver stiffness reductions observed after 48 weeks at 2 mg and 10 mg doses. Patients who showed suboptimal virologic responses to bulevirtide at week 24 also benefited from continued therapy, and 43% of non-responders and 82% of partial responders achieved virologic response at week 96. Biochemical improvement was also observed and often occurred independently of virologic response, along with liver stiffness from week 48, with both the 2 mg and 10 mg treatment. Most adverse events were mild, and none were considered related to bulevirtide [23].

A short-term combination study (MYR202, a randomized, multicenter, open-label phase 2 trial with parallel groups) compared, for 24 weeks, a combination of tenofovir with daily subcutaneous bulevirtide (doses of 2 mg, 5 mg, or 10 mg) with tenofovir alone. The study included adults (aged 18 to 65 years) with chronic hepatitis delta (CHD) virus infection, encompassing patients with cirrhosis and those who had contraindications or did not respond to peginterferon therapy. Levels of HDV RNA decreased by a minimum of 2 log10 IU/mL or became undetectable in 50–77% of patients treated with bulevirtide plus tenofovir, compared to only 4% of those treated with tenofovir monotherapy [24].

Bulevirtide alone had a very low impact on the levels of HBsAg. Therapy discontinuation was followed by a rebound in HDV RNA load. The data suggest that extended combination therapy with tenofovir may be an option and should be studied, but it is not known whether long-term virologic efficacy is maintained, how long treatment should be maintained, or whether rebound still happens after or during treatment. Treatment-induced increases in bile acid levels raise safety concerns about the long-term administration of bulevirtide therapy [24].

In a long-term combination study (MYR204, a multicenter, open-label, randomized, phase 2b trial), bulevirtide was provided to two groups of participants at doses of 2 mg and 10 mg in combination with pegylated interferon alfa-2a (fixed dose of 180 µg per week) for 48 weeks, followed by the same daily dose of bulevirtide monotherapy for 48 weeks; pegylated interferon alfa-2a (180 µg per week) alone for 48 weeks or bulevirtide alone (10 mg sc per day) was continued for 96 weeks [25]. The primary outcome was defined as undetectable levels of HDV RNA 24 weeks after completion of treatment. The principal comparison evaluated the efficacy of 10 mg bulevirtide combined with peginterferon alfa-2a versus the 10 mg bulevirtide alone group. Analysis at 24 weeks post-treatment showed that HDV RNA was undetectable in 17% of the participants in the peginterferon alfa-2a group, 32% of those in the 2 mg bulevirtide plus peginterferon alfa-2a group, 46% in the 10 mg bulevirtide plus peginterferon alfa-2a group, and 12% in the 10 mg bulevirtide group alone. At 48 weeks after treatment, HDV RNA remained undetectable in 25% of the participants in the peginterferon alfa-2a group, 26% in the 2 mg bulevirtide plus peginterferon alfa-2a group, 46% in the 10 mg bulevirtide plus peginterferon alfa-2a group, and 12% in the 10 mg bulevirtide group. Once again, the results from combination therapy and higher doses of bulevirtide are better than bulevirtide standard alone [25].

In patients receiving bulevirtide, peginterferon alfa-2a significantly accelerated the kinetics of viral decline. The estimated efficacy of bulevirtide in preventing hepatocyte infection was estimated at 90.3%, whereas peginterferon alfa-2a inhibited viral production with 92.4% efficacy, despite considerable inter-individual variability. According to mathematical modeling, treatment extended to 144 weeks may increase viral cure rates to 42.1% with bulevirtide monotherapy and 66.7% with bulevirtide plus peginterferon alfa-2a [26]. Prospective clinical trials are necessary to confirm these findings. Bulevirtide inhibits the extracellular spread of HBsAg-dependent virion propagation, and interferon inhibits HBsAg-independent propagation mediated through cell division, probably explaining the therapeutic synergism observed [7]. Bulevirtide treatment strongly reduces intrahepatic HDV RNA levels, the proportion of HDV-infected hepatocytes, and the level in viremia, without reducing the intrahepatic levels of HBV. This seems to be enough to diminish the signs of liver inflammation observed in the long-term bulevirtide treatment, suggesting that blocking viral entry ameliorates liver inflammation signs and that prolonged therapeutic regimens may result in HDV eradication in a proportion of patients. These findings provide an important proof of concept that will underpin the design of treatment strategies and combination therapies in individuals with chronic delta hepatitis [27].

When patients present with detectable HDV viremia, liver fibrosis without decompensation, and no contraindications should receive pegylated interferon alfa-2a, especially in regions where bulevirtide is unavailable.

### 7.2. Lonafarnib

Lonafarnib (LNF) is a drug that is already commercially available and used as a therapeutic approach for Hutchinson-Gilford progeria syndrome (which causes children to age rapidly, starting in their first two years of life). Lonafarnib, a farnesyl-transferase inhibitor, effectively inhibits HDV particle assembly and secretion. Prenylation is a critical step in the HDV life cycle, and its blockade abolishes HDV production in experimental models. Lonafarnib is an oral first-in-class prenylation inhibitor, with proven efficacy in individuals infected with HDV.

In an exploratory proof-of-concept trial, 200 mg of lonafarnib was administered orally twice daily as monotherapy for 4 weeks, with patients followed for 6 months. A mean decline in HDV RNA of 1.54 log10 IU/mL relative to baseline was observed. Lonafarnib serum concentrations were associated with changes in HDV RNA levels. There was no evidence of virological resistance. However, all patients (100%) experienced weight loss and gastrointestinal secondary effects, including nausea, abdominal bloating, and diarrhea [28]. These results were further improved by combining lonafarnib with ritonavir plus pegylated interferon alfa (LOWR HDV-1 study). The combination of a lower-dose lonafarnib (100 mg twice daily) with ritonavir (100 mg QD), a CYP 3A4 inhibitor, produced superior antiviral responses compared to a higher dose of lonafarnib alone while causing fewer side effects [29].

In the LOWR HDV-2 trial, a single-center, open-label, phase 2 dose-finding study, a combination of high lonafarnib doses (75 mg administered orally twice daily with ritonavir (12 weeks) and low doses of lonafarnib (25 or 50 mg orally twice daily) combined with ritonavir with or without peginterferon (24 weeks) alfa were assessed. The primary efficacy endpoint, defined as at least a 2 log10 or greater decrease or undetectable quantification of HDV viremia from baseline at end of treatment, was achieved in 46% of patients (6/13) treated with the all-oral regimen (lonafarnib 50 mg, po, twice daily plus ritonavir) and 89% (8/9) treated with combination regimens (lonafarnib, 25 or 50 mg twice daily with ritonavir plus peginterferon alfa). Several patients experienced brief post-treatment ALT elevations, which were associated with undetectable blood HDV RNA and normalization of ALT levels [30].

Lonafarnib, when combined with low-dose ritonavir, represents a promising oral treatment option, with its greatest effectiveness observed when peginterferon alfa is added. The identification of optimal regimens justified a phase 3 clinical trial of LNF for HDV. To study the efficacy and safety of ritonavir-boosted lonafarnib, with or without peginterferon alfa, the phase 3 D-LIVR study enrolled 407 individuals diagnosed with compensated liver disease linked to CHD being treated with NA therapy who had achieved HBV DNA suppression for at least 12 weeks, and presented with elevated serum ALT levels (>1.3 × ULN and <10 × ULN). Participants were randomly allocated into four arms: group 1 received lonafarnib (50 mg twice daily) with ritonavir, group 2 received lonafarnib plus ritonavir and pegIFN-α, group 3 received pegIFN-α monotherapy, and group 4 received a placebo. The therapeutic regimen lasted 48 weeks, after which patients underwent a 24-week observation phase without treatment. Paired liver biopsies were obtained at week 48.

At treatment completion, virologic response rates were 14.6%, 32%, 36.5%, and 3.8% in the respective groups. Biochemical responses were reported in 24.7%, 34.4%, 11.5%, and 7.7%, while combined response rates reached 10.1%, 19.2%, 9.6%, and 1.9%. An improvement of more than two points in the histologic activity index without worsening of fibrosis was achieved in group 1 (33%), group 2 (53%), group 3 (38%), and group 4 (27%) of patients. Lonafarnib was well tolerated, and the safety profile was consistent across all treatment groups [31].

Following phase 3 studies, LNF received orphan drug designation in the US and Europe. Its efficacy appears largely limited to HDV RNA suppression, with little or no effect on the levels of HBsAg [31].

### 7.3. Nucleic Acid-Based Therapies

Three classes of molecular compounds are being investigated for their potential in the treatment of HBV and HDV infections. Therapeutic strategies involving nucleic acids include nucleic acid polymers (NAPs), small interfering RNAs (siRNAs), and antisense oligonucleotides (ASOs) [32]. Nucleic acid polymers (NAPs) act in the later steps of the hepatitis B replication cycle, disrupting the assembly and release of HBV VLPs (which constitute >99.99% of circulating HBsAg) and blocking the release of HBsAg. VLPs have important immune-inhibitory effects, which hinder the restoration of immune control and the achievement of a functional cure [32,33].

REP 2139 is a NAP that led to high HBV/HDV suppression rates. Through interaction with host chaperone proteins, it inhibits the assembly and release of HBsAg [34]. When combined with peginterferon alfa-2a and tenofovir, it is safe after one year of therapy and induces clinical control of HBV and HDV coinfection with normalization of serum aminotransferase levels in a substantial proportion of individuals.

In the REP 301 trial, a phase 2 study, twelve untreated, HBeAg-negative patients without cirrhosis, CHD, and high HBsAg levels (>1000 IU/mL), were enrolled to receive treatment with REP 2139-Ca (calcium chelate complex formulation). The regimen consisted of 15 weeks of weekly intravenous infusions of 500 mg REP 2139-Ca, followed by 250 mg REP 2139-Ca combined with peginterferon alfa-2a 180 mcg weekly for 15 weeks and peginterferon alfa-2a alone for 33 weeks. By the conclusion of treatment, 50% of participants achieved HBsAg clearance, with five maintaining negativity at one year. Anti-HBs seroconversion was observed in five individuals and maintained at follow-up. HDV RNA levels dropped below detectable limits in eleven patients during therapy, with sustained virologic response observed in seven individuals at one and 3.5 years follow-up [35,36].

ASOs are short, single-stranded synthetic RNA or DNA molecules, while siRNAs are double-stranded RNA sequences referred to as siRNA [32,33].

ASOs hybridize directly with their target RNA through complementary base pairing, inducing either RNA degradation or translational inhibition. siRNAs associate with the RNA-induced silencing complex (RISC), where a single siRNA strand is selected to serve as the guide. The guide strand enables RISC to identify and attach to complementary sequences within messenger RNA or viral RNA, resulting in their targeted cleavage and subsequent degradation. By targeting RNA molecules, both ASOs and siRNA are engineered to silence specific genes and degrade HBV mRNA, thereby preventing protein synthesis and stopping the production of HBV proteins, particularly HBsAg [32,33].

Elebsiran (VIR-2218), a siRNA, was assessed alone and combined with peginterferon alfa-2a. The treatment produced concentration-dependent reductions in HBsAg levels; HBsAg clearance was not achieved in any patients treated solely with elebsiran [37]. In the SOLSTICE trial, a phase 2 study, elebsiran was further evaluated in combination with tobevibart (VIR-3434), a monoclonal antibody targeting HBsAg, in patients with CHD. By week 24, all patients achieved a virologic response, with HDV RNA becoming undetectable in 41% of participants, rising to 80% in a subgroup treated for 60 weeks [38].

JNJ-3989 (JNJ-73763989), another siRNA, was engineered to silence the transcriptional activity of all forms of HBV RNAs, comprising both messenger and pregenomic RNA, thereby inhibiting the synthesis of HBV viral proteins. The REEF-D proof-of-concept study evaluated its therapeutic efficacy in patients with CHD. In the initial cohort, 17 participants received JNJ-3989 combined with a nucleos(t)ide analog (NA). By week 48, four demonstrated both virologic and biochemical responses, but treatment was discontinued in eight individuals due to persistent HBsAg and rising ALT, predominantly in those with high baseline levels of HBsAg (>10,000 IU/mL) together with HDV RNA (>100,000 IU/mL) [39,40]. In a second study phase, cirrhotic patients or those with high baseline levels of HBsAg and HDV RNA were excluded. The trial is planned for 144 weeks. Interim data reported at week 48 showed that among 15 patients treated with JNJ-3989, 7 (47%) attained a virologic response while maintaining normal ALT values. ALT elevations occurred in 29% (7 of 24 patients), 6 of whom had high baseline HBsAg. When data from both 1 and 2 phases were combined, 52% (14 of 27) of the patients attained a virologic response by week 48, with 11 also showing a biochemical response. Patients with high HBsAg levels at baseline all developed ALT increases during therapy [41].

An ASO, Bepirovirsen (GSK3228836), targeting HBV mRNAs stands out as one of the most promising in its class. In the short term (week 24), it is able to produce a sustained HBsAg loss and HBV DNA reduction in 9–10% of individuals, regardless of NA [42].

### 7.4. Antibody-Based Therapies

Tobevibart, a monoclonal antibody targeting HBsAg (antigenic loop), demonstrated broad neutralizing capacity across all tested genotypes in preclinical models. Preclinical data, along with interim phase 1 study results, confirmed superior neutralizing activity compared to hepatitis B immunoglobulin (HBIG) against HBV and HDV [43].

In the SOLSTICE phase 2 trial, tobevibart was investigated as monotherapy (300 mg biweekly) and combined with elebsiran (VIR-2218), a siRNA (200 mg every month). The elebsiran component reduced extracellular HBsAg and HDV particles, while tobevibart displayed broad genotypic neutralization. The combination produced additive reductions in both HBV and HDV levels [44]. Clinically, combination therapy achieved higher efficacy than monotherapy, with all patients reaching virologic response with a high proportion of HDV RNA suppression at week 24 that rises with longer treatment duration [38]. Monotherapy and higher baseline HDV RNA correlated with lower response probability. For combination therapy, cirrhotic status, viral load, and HBsAg levels were not predictive of outcome [45].

Brelovitug (BJT-778), another monoclonal antibody anti-HBsAg, is under evaluation in a phase 2 trial for chronic hepatitis delta. Three dosing schedules are being tested over a 48-week study period as follows: group 1: administration of 300 mg per week, group 2: 600 mg per week for 12 weeks, followed by the same dose biweekly, and group 3: 900 mg biweekly before switching to monthly dosing. Interim data from groups 1 and 2 showed encouraging results. In group 1, 7 out of 10 patients demonstrated a virologic response at week 12, defined as a ≥2 log10 IU/mL reduction in HDV RNA from baseline or HDV RNA bellow the limit of detection, 5 out of 9 showed a combined response, and 1 out of 10 had undetectable HDV RNA; by week 28, virologic response was universal, with 6 out of 9 combined responses and 6 out of 10 achieving undetectable HDV RNA levels. In group 2, 8 out of 10 achieved a virologic response, 2 out of 4 showed combined responses, and 3 out of 10 were undetectable RNA for HDV at week 12. Participant enrollment is currently underway for AZURE-1, a global phase 2b/3 clinical trial [46].

Libevitug (HH003) is an innovative monoclonal antibody directed at the pre-S1 segment of L-HBV, thereby preventing viral entry and reinfection of hepatocytes. Preclinical data indicated activity toward HBV and HDV viruses. In an open-label, phase 2 trial of nine adults, the antibody demonstrated favorable safety and tolerability, alongside strong antiviral activity. Libevitug treatment led to significant reductions in HDV RNA levels and normalization of ALT values. Group 1 received Libevitug at 20 mg/kg intravenously every two weeks with tenofovir alafenamide (TAF) for 48 weeks, group 2 received 10 mg/kg on the same schedule, and the control group received TAF alone. All participants remain in follow-up. HH003 has been granted Breakthrough Therapy Designation (Nov 2024), and its phase 2b trial (NCT05861674) has completed the treatment phase [47].

GIGA-2339, the first human recombinant polyclonal antibody of its kind, comprises a pool of distinct IgG clones that target HBsAg. These antibodies bind to multiple epitopes, demonstrating the capacity to neutralize HDV genotype 1 virions enveloped by HBsAg derived from each of the HBV genotypes A–H. It is expected to maintain efficacy across all HBV genotypes and against a broad range of circulating HBV variants, including potential escape mutants, potentially allowing for viral clearance and long-term immune system activation.

Preclinical research and data from an ongoing phase 1 trial showed that the capacity of neutralization was greater (more than 2000 times) than plasma-derived HBIG. Other properties, such as enhanced antibody-dependent cellular cytotoxicity and antibody-dependent cellular phagocytosis, were greater than the comparator (tenfold) HBIG. Collectively, these findings highlight the promise of this approach as a future therapeutic strategy for the treatment of HBV and HDV infections [48].

## 8. Liver Transplantation

Chronic HDV infection has an accelerated course. Patients with HDV-related cirrhosis are generally younger than those with HBV- and HCV-related cirrhosis and progress more rapidly to decompensated liver disease. Consequently, due to the rapid progression to end-stage liver disease, death is more often caused by liver failure than by hepatocellular carcinoma, which may not have sufficient time to develop [49].

Compared to patients with undetectable HDV RNA, those with detectable HDV RNA had a significantly higher risk of progression to cirrhosis, hepatocellular carcinoma, and an increased likelihood of liver transplantation and liver-related mortality. The rapid progression and the lack of effective therapy mean that liver transplantation remains a treatment option for a significant proportion of patients with HDV infection. In HDV-infected patients with decompensated cirrhosis or fulminant hepatitis, the use of interferon is limited due to adverse effects. In these settings, the only treatment option is liver transplantation. Patients undergoing orthotopic liver transplantation for HDV-related cirrhosis experience favorable long-term outcomes, with 5-year survival rates around 88% [50]. Subjacent HBV infection is an important determinant of the recrudescence after liver transplantation, and the rate of graft reinfection occurs less frequently in HDV than in HBV transplant recipients. The often undetectable or low HBV DNA viral load at the time of liver transplantation, combined with prophylaxis against HBsAg using HBIG, further reduces the risk of HDV reinfection of the graft. The pretransplant viral load of HDV RNA in the blood has no prognostic relevance because the risk of transmission is linked only to the HBV viremia. The graft may be infected with HBV-HDV coinfection, meaning that HBV infection is manifested first in the liver graft, allowing the secondary expression of the HDV [51].

Combined prophylaxis with nucleosid(t)e analogs against HBV with HBIG is now standard prophylaxis; it leads to a decrease to 0–10% of the recurrence at 1–2 years post-transplantation and to a reduced dose of HBIg required in the long term [52].

## 9. Conclusions

The global prevalence of HDV infection remains unknown, and it may affect between 12 and 72 million people distributed across large areas of the globe in Africa, Asia, and South America. Europe also has some hot spots for hepatitis delta infection in Romania and Moldova. In developed countries, the prevalence of delta infection in human immunodeficiency virus-infected patients is similar to the general population, but HBsAg-positive PWID have a very high prevalence of hepatitis delta infection.

In HBV-HDV coinfection, progression to cirrhosis, liver failure, and hepatocellular carcinoma is more rapid than in HBV mono-infection, putting these patients at risk for premature death. Integrated systematic screening for HBV markers, HDV antibodies, and RNA HDV is the best way to diagnose all cases of HBV-HDV coinfection.

Medical treatment has traditionally relied on the use of interferon alfa and has been the only off-label and nonspecific option to treat chronic HDV-HDV hepatitis for years, but after 48 weeks of treatment, the efficacy was very low regarding sustained virological response and HBsAg clearance. Bulevirtide is now the only approved treatment against hepatitis delta. The results of efficacy studies are based on the combined HDV viremia and biochemical responses, but their clinical relevance is currently unknown. Combination therapies of bulevirtide with interferon or nucleos(t)ide analogs have greater efficacy than monotherapy alone. Other novel drugs targeting different stages of the HDV replication cycle are currently under investigation, showing promising results.

Surgical treatment is reserved for end-stage liver disease, but without prophylaxis, sequential reinfection of the graft by HBV followed by HDV was the rule. Nucleosid(t)e analogs against the HBV combined with hepatitis B immunoglobulin is now the standard prophylaxis that has resulted in a significant reduction in post-transplant HBsAG recurrence.

## Figures and Tables

**Table 1:** Advances in therapeutic strategies for HDV infection.

Class	Drugs/Regimens	Route	Drug Sponsor	Mechanism of Action	Development	* Doses/Regimens
Immunomodulators	PegIFN-α-2a/−2b	SC	PharmaEssentia	Immune activation	Approved off-label HDV	180 mcg qwk/1.5 mcg/kg qwk − 48 wk
Immunomodulators	PegIFN-λ	SC	Eiger InnoTherapeutics	Immune activation, Type III IFN	Phase 2/3 (stopped)	120 to 180 mcg qwk − 48 wk
Entry inhibitors	Bulevirtide	SC	Gilead	NTCP receptor inhibition	** Approved 2 mg qd	2 to 10 mg qd
Entry inhibitors	Bulevirtide + PegIFN-α	SC	–	NTCP receptor inhibition + immune activation	Phase 2	2 to 10 mg qd + PegIFN-α − 96 wk
Assembly/secretion inhibitors	Lonafarnib + Ritonavir	PO	Eiger InnoTherapeutics	Inhibition of L-HDAg prenylation	Phase 3	50 mg BID/Ritonavir − 48 wk
Assembly/secretion inhibitors	Lonafarnib + Ritonavir + PegIFN-α	PO + SC	–	Inhibition of L-HDAg prenylation + immune activation	Phase 3	50 mg BID/Ritonavir + PegIFN-α − 48 wk
Nucleic acid-directed therapies	Elesbiran + Tobevibart	SC	Vir Biotechnology	siRNA (HBV mRNA silencing) + HBsAg targeting mAb (HBsAg neutralization)	Phase 2–3	100/200 mg q1m + Tobevibart − 48 wk
Nucleic acid-directed therapies	JNJ-3989	SC	GlaxoSmithKline Pharmaceuticals	siRNA (all HBV RNA transcription silencing)	Phase 2	100 mg q4 wk + NA − 144 wk
Nucleic acid-directed therapies	REP 2139-Ca/-Mg	IV	Replicor	NAP (binds host chaperones + blocks assembly and HBsAg secretion)	Phase 2	REP 2139-Ca − 500 mg IV qwk 15 wk, then 250 mg IV qwk + PegIFN-α 15 wk (until 33 wk) REP 2139-Mg − 250 mg IV qwk ± PegIFN-α, for 48 wk
Antibody-based therapies—Monoclonal	Tobevibart	SC	Vir Biotechnology	anti-HBsAg (HBsAg neutralization)	Phase 2/3	300 mg q2 wk
Antibody-based therapies—Monoclonal	Brelovitug	SC	Bluejay	anti-HBsAg (HBsAg neutralization)	Phase 2b/3	300 mg qwk or 900 mg q1m
Antibody-based therapies—Monoclonal	Libevitug	IV	HuaHei	Pre-S1 domain (HBsAg neutralization)	Phase 2	20 mg/kg q2wks − 24 wk, and 24 wk follow-up
Antibody-based therapies—Monoclonal	RG-6349; 2H5-A14, E6F6; H3B-6520; CM1239(20)	SC	–	Anti-HBsAg; preS1-NTCP block; HBsAg clearance; direct NTCP block	Phase 1	—
Antibody-based therapies—Polyclonal	GIGA-2339	IV	IgaGen	Anti-HBsAg (broad neutralization)	Phase 1	—

Abbreviations (in alphabetical order): BID = twice daily; ** EMA = European Medicines Agency; HBsAg = hepatitis B surface antigen; HDV = hepatitis delta virus; IV = intravenous route; kg = kilogram; L-HDAg = large hepatitis delta antigen; mcg = micrograms; NA = nucleoside/nucleoside analog; NAP = nucleic acid polymer; NTCP = sodium taurocholate cotransporting polypeptide; PegIFN = pegylated interferon; PO = per os; qd = once a day; qwk = once a week; q2wk = biweekly; q1m = once a month; SC = subcutaneous injection; siRNA = small interfering RNA; wk = week. * Most extensively evaluated treatment regimens.

## Data Availability

No new data were created or analyzed in this study. Data sharing is not applicable to this article.
